# Nonconjugated Polyurethane Derivatives with Aggregation-Induced Luminochromism for Multicolor and White Photoluminescent Films

**DOI:** 10.1021/acsmacrolett.4c00534

**Published:** 2024-09-09

**Authors:** Nan Jiang, Ya-Jie Meng, Chang-Yi Zhu, Ke-Xin Li, Xin Li, Yan-Hong Xu, Jia-Wei Xu, Martin R. Bryce

**Affiliations:** † Key Laboratory of Preparation and Applications of Environmental Friendly Materials, Key Laboratory of Functional Materials Physics and Chemistry of the Ministry of Education, Jilin Normal University, Changchun 130103, China; ‡ Ministry-of-Education Key Laboratory of Numerical Simulation of Large-Scale Complex System (NSLSCS) and School of Chemistry and Materials Science, Nanjing Normal University, Nanjing 210023, China; § Department of Chemistry, Durham University, Durham DH1 3LE, United Kingdom

## Abstract

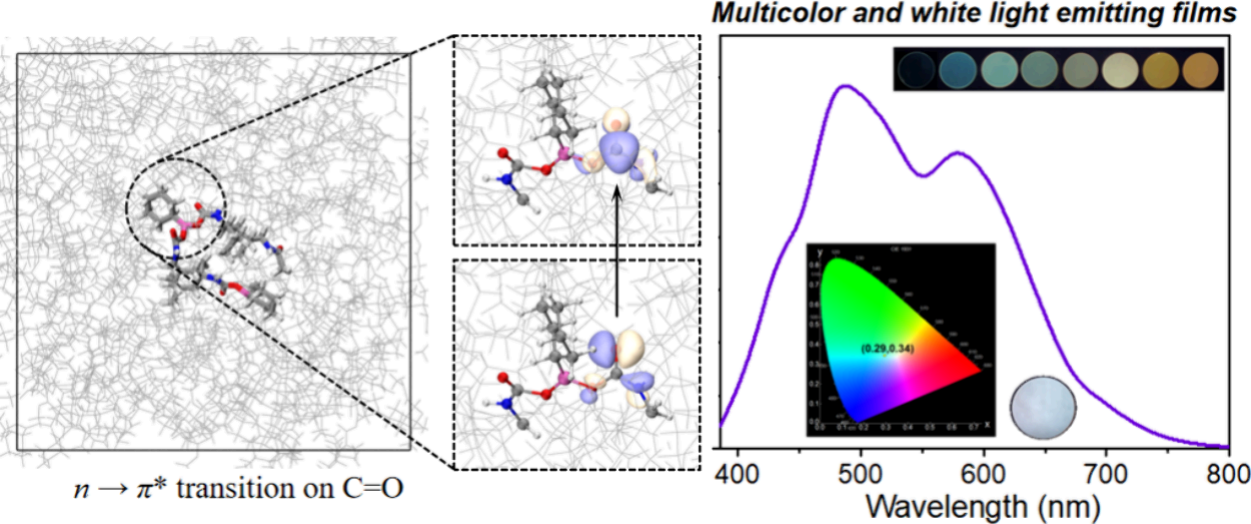

A simple and effective strategy to obtain solid-state multicolor emitting materials is a particularly attractive topic. Nonconventional/nonconjugated polymers are receiving widespread attention because of their advantages of rich structural diversity, low cost, and good processability. However, it is difficult to control the molecular conformation or to obtain the crystal structure of amorphous molecules, which means it is a challenge to obtain nontraditional polymeric materials with multicolor emission. In this work, a polyurethane derivative (**PUH**) with red-shifted emission was synthesized by a simple one-pot polymerization reaction. By exploiting the aggregation-induced luminochromism of **PUH**, a series of plastic films with tunable emission from blue to orange, and white-light emission, was obtained by doping different amounts of **PUH** into poly(methyl methacrylate) (PMMA), thereby changing the aggregation degree of **PUH**. This work demonstrates the excellent promise of polyurethane derivatives for the simple fabrication of large-scale flexible luminescent films.

Traditional photoluminescence relies on the excitation of the delocalized electrons within aromatic or extended π-systems.^1,2^ However, these discrete chromophores often face the dilemma of concentration-dependent emission quenching, resulting in a significant reduction in the emission intensity of solid/aggregated samples, which greatly limits their photoluminescent applications.^3−7^ Since the 1970s, the emergence of nonconventional luminescent materials has overcome this problem.^8−11^ It has been found that some organic small molecules, macromolecules, inorganic, and organic/inorganic hybrid materials without traditional fluorophores also exhibit inexplicable and nonconventional intrinsic luminescence properties.^12−14^ Unlike traditional chromophores, nonconventional chromophores do not require (hetero)aromatic π-conjugated structures, and they work via the collective intra- and intermolecular associations and the subsequent through-space electronic conjugation of nonconjugated subunits to achieve emission.

These so-called nonconventional chromophores comprise hydroxyl (−OH), sulfur (−S−), ester (−COOR), carboxyl (−COOH), carbonyl (C=O), alkene (C=C), ether (−O−), and amide (−NHCO−) units and others.^15,16^ However, due to the lack of large π-conjugated units, these nonconventional luminescence are mostly limited to blue emission regions. Enhancing the through-space electronic communication and conjugation of materials by polymerization has been recognized as an effective strategy for regulating nontraditional luminescence.^17−20^ Nonconventional luminescent polymers (NCLPs) have emerging applications in anticounterfeiting, sensor and photoelectric devices, clinical therapy, and other fields.^21−23^

The optimal molecular design principles of NCLPs with longer wavelength emission are still being explored.^24−28^ Strong and stable interchain and/or intrachain through-space interactions (TSIs) are ways to facilitate strong, red-shifted emission.^29^ Effective TSIs require an appropriate balance of the rigidity and flexibility of the polymer chains. For example, Zhang et al. used different anhydrides to manipulate segmental flexibility and rigidity of nonconjugated and nonaromatic polyesters, and obtained variable (yellow-green) luminescence.^30^ Wang et al. synthesized poly(itaconic anhydride-*co*-vinyl caprolactam) with orange–red emission, emphasizing that a suitable molecular chain conformation is conducive to stronger intrachain and/or interchain interactions as the decisive factor in determining the emission of these NCLPs.^31^ Nonaromatic poly(maleimides) provide controllable blue-to-red emission.^32^ These outstanding examples are precedents for tuning the long-wavelength emission of unconventional materials. However, monotonic luminescence modulation usually does not meet practical application requirements. Nonconventional chromophores with adjustable multicolor luminescence have also been studied. For instance, Cloutet et al. established the importance of oxygen aggregation (caused by the restricted polymer conformation) for the multichromatic photoluminescence of poly(dihydropyran).^33^

Inspired by the above background, we have now synthesized a nonconjugated polyurethane derivative with an unsealed end structure using cyclohexylboronic acid and isophorone diisocyanate monomers. The cyclohexyl and isophorone rings bring greater rigidity to the polyurethane skeleton as opposed to flexible linear alkyl chain monomers. Polyurethane is an organic polymer intermediate between plastics and rubber.^34^ Because of its favorable mechanical properties, excellent resistance to climate, chemical corrosion, fatigue, wear, and high impact resistance, it has become one of the most commonly used polymers in everyday life and in industry.^35−37^ However, research on polyurethane derivatives as luminescent materials is often ignored. The repeating urethane unit [−NHC(O)O−] is an ideal multiheteroatomic template for exploring nontraditional luminescence phenomena with excellent application potentials.^38−41^

This work describes a luminescent polyurethane derivative (**PUH**) with long-wavelength emission, which is the result of the appropriate rigid, distorted molecular structure and electron-rich repeat units. In addition, using the luminescence characteristics of **PUH**, a series of films with a fluorescent gradient were prepared by adjusting the doping ratio of **PUH** and poly(methyl methacrylate) (PMMA) to regulate the extent of aggregation of the polymer, finally, to obtain transparent plastic films with multicolor (blue to orange) and white luminescence. This work provides experimental and theoretical validation for the design strategy of long-wavelength emitting NCLPs and serves as a new case study for their potential practical applications.

Through simple one-pot polymerization reactions, multicolor fluorescent polyurethane derivatives were obtained (Figure 1a, Tables S1 and S2), named **P1**, **P2**, **P3**, **PUC**, and **PUH** (Figures S1 and S2). ^1^H NMR and FT-IR spectra (Figures S3−S9) show that the PUs are well-structured materials. The absorption and emission spectra showed that the PUs exhibited different light utilization and a blue to orange-red luminescence, respectively, under 365 nm ultraviolet excitation (Figures 1b and S10). Since the PUs’ structure does not contain any conventional chromophore, their luminescence is ascribed to polymerization-induced nonconventional intrinsic luminescence.

**Figure 1:**
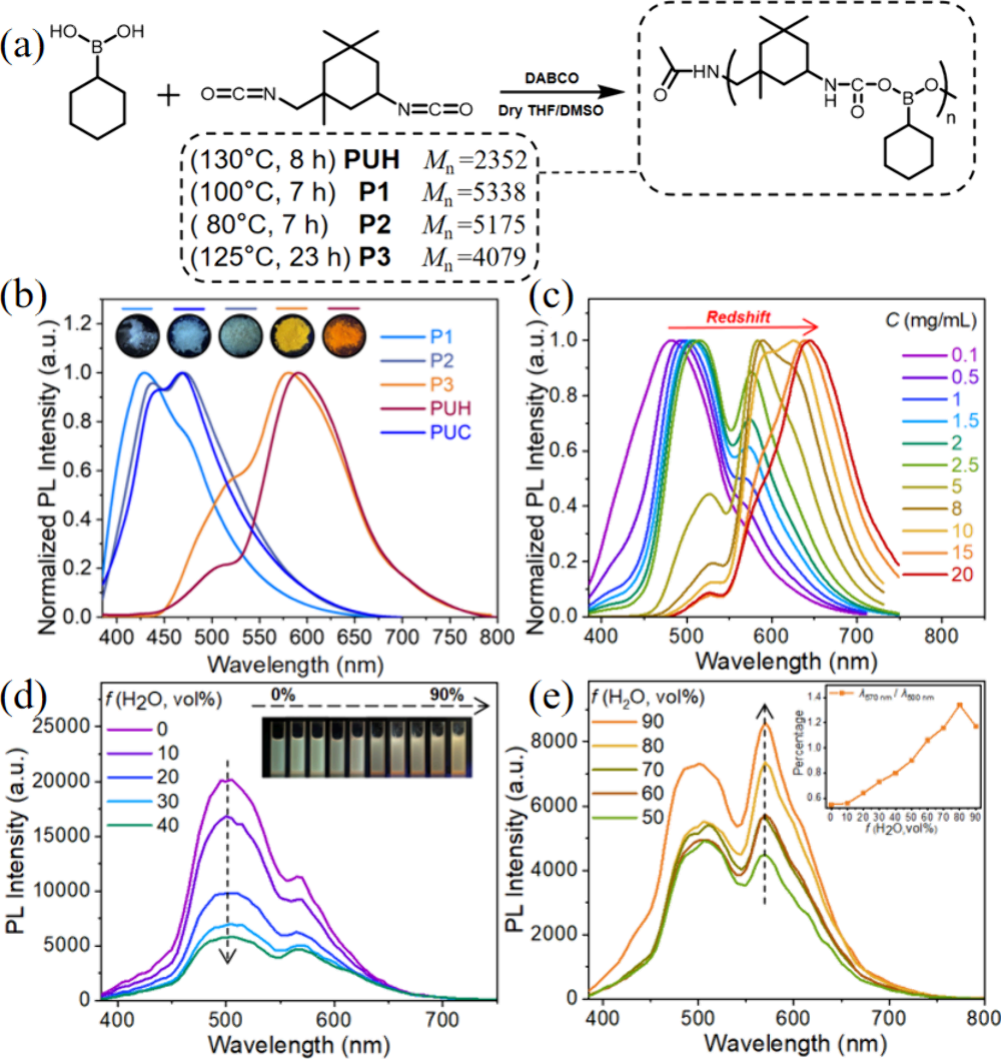
(a) Synthetic method and chemical structures of **PUH**, **P1**, **P2**, and **P3**, and the corresponding average molecular weights. (b) Normalized emission spectra of **P1**, **P2**, **P3**, **PUH**, and **PUC** powder (λ_ex_ = 365 nm). Insets: the corresponding fluorescence photographs under 365 nm UV illumination. (c) Normalized emission spectra of **PUH**/DMSO with different concentrations. (d, e) Emission spectra of **PUH** (1 mg mL^–1^) in DMSO–water mixtures with different water fractions (0–90% v/v) at room temperature. Insets: corresponding photographs under 365 nm UV illumination, and the intensity ratio plot of λ_570 nm_/λ_500 nm_ with the increase of water content.

Taking **PUH** which has the reddest emission as the main focus, the emission spectra of **PUH**/DMSO solutions at different concentrations revealed a large redshift of 164 nm for the main peak with the gradual increase of concentration from 0.1 to 20 mg mL^–1^, with aggregation behavior of **PUH** causing the changes from green to orange emission (Figure 1c). Such wide variations in emission peaks as observed here with **PUH** are rare,^3,42,43^ which suggests its potential for multifunctional applications. Steady-state fluorescence spectra in different solvents showed that there is charge transfer from electron donor to electron acceptor (Figure S11) in the **PUH** system, which is conducive to its luminescence.^44−46^

The aggregation process of **PUH** was probed by scanning electron microscopy (SEM). Initially, the cyclohexylboronic acid monomer has a sparse irregular structure; however, after polymerization, at the same magnification, **PUH** presents a very aggregated nanospherical structure (Figure S12). It can be inferred that this progressive aggregation behavior caused by polymerization is closely related to the change in the materials’ photophysical properties. As shown in Figures 2 and S13, the microstructure of the **PUH** solutions was monitored at different concentrations via SEM and dynamic light scattering (DLS). Even at low concentrations, **PUH** showed a relatively concentrated structure compared to that of the monomer, except with a smaller particle size (Figure 2a). As the concentration of the solution increased, **PUH** gradually accumulated and slowly transformed into a large nanosized spherical aggregate structure (Figures 2b–d and S13). This change in the microscopic structure correlates positively with the concentration-dependent emission data in Figure 1c.

**Figure 2:**
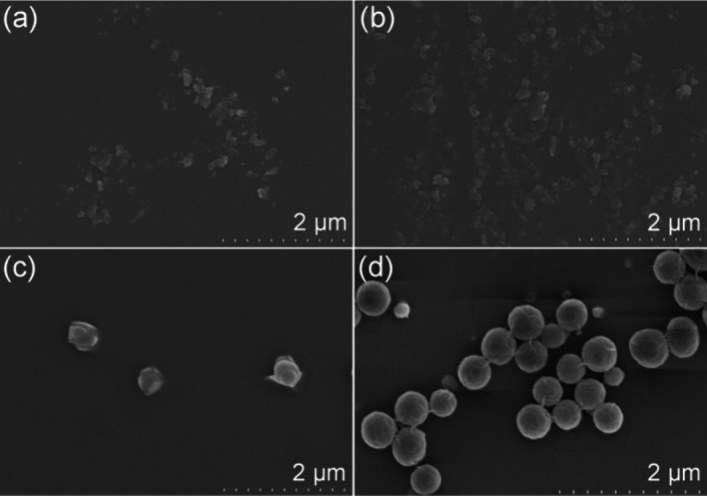
SEM images of **PUH**: (a) 0.1 mg mL^–1^, (b) 1 mg mL^–1^, (c) 5 mg mL^–1^, and (d) 10 mg mL^–1^ in pure DMSO solution.

The emission spectra of solid powders of PUs at different excitation wavelengths are presented in Figures S14 and S15. **PUH** and **P3** with longer wavelengths of emission have weaker excitation-dependent emission characteristics. The SEM results showed that **P3** had a compact microscopic aggregation structure (Figure S16) similar to that of **PUH** (Figure 2). It is assumed that the high reaction temperature increases the molecular aggregation. The microstructures of **P1** and **P2** are relatively loose. It is speculated that compact aggregation is not only beneficial to obtain long wavelength emission, but also to obtain luminous clusters with uniform electronic delocalization.

1,4-Cyclohexanediol monomer was selected for comparative experiments. Even if the reaction temperature and time were increased to 140 °C and 21 h, product **PUC** still showed only blue fluorescence. Therefore, the importance of the boron atom in the long-wavelength emission in this system is confirmed. This will be discussed in detail in the theoretical calculations section.

Obvious aggregation-induced luminous discoloration was also observed in the mixed DMSO/water solutions of **PUH** (Figure 1d,e). When **PUH** is dissolved in the good solvent DMSO, due to the low concentration (0.1 mg mL^–1^) the **PUH** molecules are in a sparsely dispersed state. The emission spectrum of the pure **PUH** solution has a main peak at 500 nm and a shoulder peak at 570 nm (Figure 1d) showing green emission under 365 nm UV light. However, with the gradual addition of adverse solvent water, the dispersed **PUH** molecules randomly rearrange and passively aggregate.^14,47,48^ The sparse molecular chains gradually aggregate into larger luminescent clusters. Thus, the emission intensity at short-wavelength (500 nm) gradually decreases, while the proportion of long-wavelength emission (570 nm) gradually increases, and the luminous color of the mixed solution gradually becomes yellow, similar to its emission in the solid state.

Unlike traditional flat luminous dyes, which rely on the formation of excimers to produce multipeak emission, polymers with nonconjugated structures often rely on soft and variable inter/intrachain entanglements to produce a variety of aggregate emission species of different sizes. These aggregates have heterogeneous electron delocalization, and thus they exhibit multipeak emissions.^49−51^ However, such multimodal emission usually has a narrow range of variation. **PUH** in the aggregated state exhibits a ratio-type multipeak emission with a large gamut variation.^32,52^ To eliminate the effect of a small amount of impurities that may exist, we mixed the cyclohexyl boronic acid monomer with **PUH** (1:1 w/w) and added the good solvent DMSO to make a solution, and then gradually added the poor solvent (water). As shown in Figure S17, the mixed solution did not produce the chromic behavior seen in Figure 1. The results suggest that the chromic behavior is derived from the self-chain aggregation of **PUH**, rather than possible impurities from the monomer. In addition, the short-wavelength emission of **P1** and **P2** (Figure 1a), which have the same chemical structure as **PUH** but different molecular weights and aggregation structures, should rule out the possibility that the polychromatic emission behavior of **PUH** is caused by the byproducts of the polymerization reaction.

Hybrid quantum mechanics/molecular mechanics (QM/MM) simulations suggested that the excitation feature for the S_1_ state of **PUH** shows an *n* → π* transition on C=O, as revealed by natural transition orbitals (Figure 3a). To understand the luminescence mechanism of **PUH**, three-stage molecular dynamics (MD) simulations, including (1) 50.0 ns equilibrium at 300 K, (2) 50.0 heating at synthetic temperature, and (3) 50.0 ns annealing to 300 K, were performed for **PUH** in both aqueous and aggregate environments. As shown in Figure 3b, in aqueous solution, the number of hydrogen bonds was not affected by the annealing process; the average number changed by only 0.16 (8.66 → 8.50). These hydrogen bonds were all formed between **PUH** and water and therefore were not sensitive to annealing, while intramolecular hydrogen bonding was almost not detected. In contrast, in the aggregate environment, the number of intermolecular hydrogen bonds showed a significant increase (60.75 → 67.40) after annealing, indicating that conformational change at a higher synthetic temperature achieved an enhanced cluster structure and contributed to the red-shift in luminescence. These results are consistent with the experimental data (Figure 1a).

**Figure 3:**
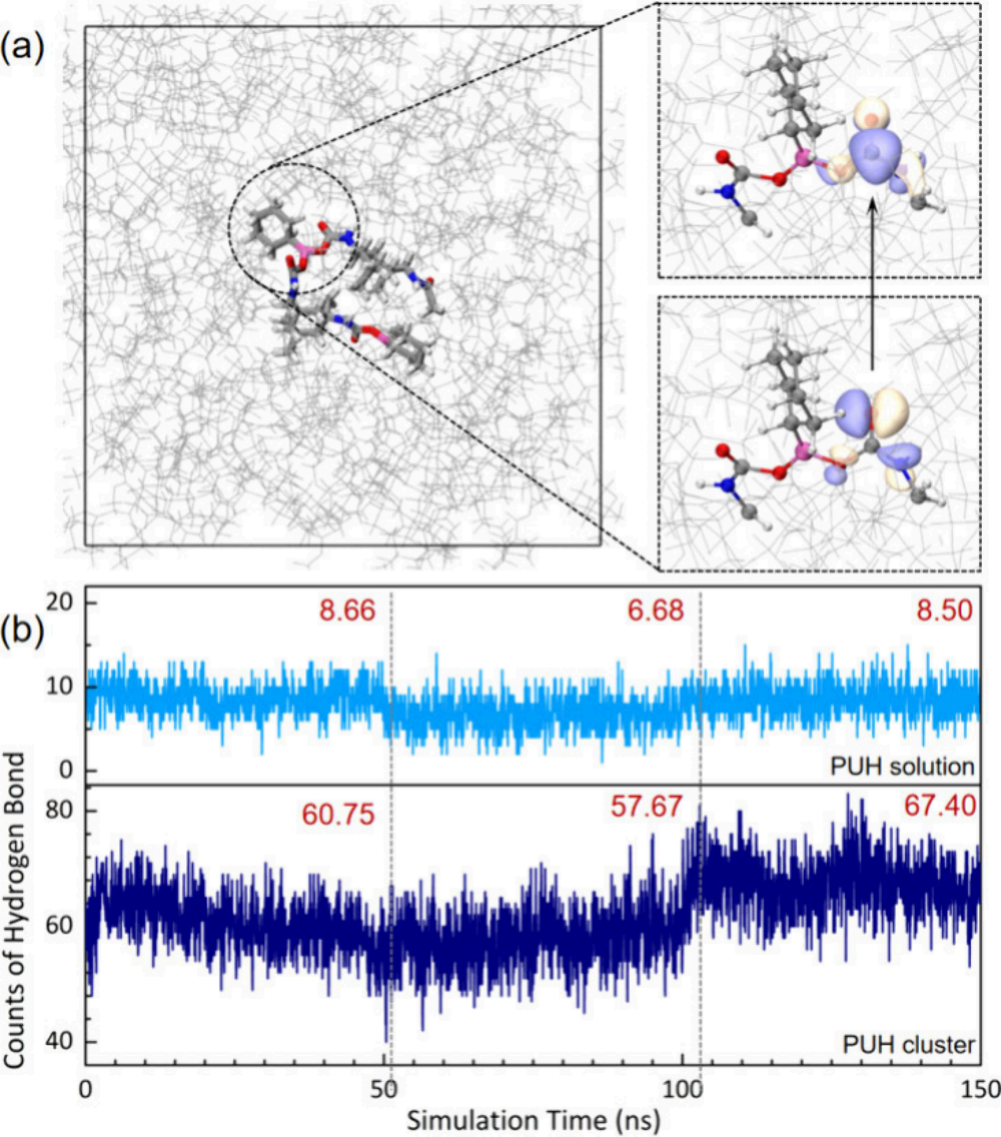
(a) Natural transition orbitals obtained from QM/MM simulations based on a snapshot taken from the MD3 trajectory. (b) Counts of hydrogen bonds during the three-stage MD simulation of **PUH** in aqueous solution (upper) and aggregate cluster environment (lower).

To further elucidate the role of the matrix environment in influencing the luminescence wavelength, the excited states of **PUH** in the gas phase, aqueous solution and aggregate environment were calculated for comparison [**PUH**, **PUH**(aq) and **PUH**(s), respectively]. As summarized in Table S3, the excitation energy of the S_1_ state was slightly decreased by aqueous solution, from 6.996 to 6.978 eV, both of which are too high to respond to common UV–vis excitation and are also unable to show luminescence within the visible region. This demonstrates that hydrogen bonds formed between **PUH** and solvent molecules are not the major factor for red-shifting the emission. However, the aggregate environment brought a significant decrease, reaching a low-lying S_1_ state with an excitation energy of only 4.609 eV excitation energy. Such an energy gap is suitable for high-efficient UV–vis excitation and visible luminescence. We can, therefore, conclude that hydrogen bonding, along with other intermolecular noncovalent interactions formed in the aggregate environment, play a significant role in assisting the generation of luminescence and increasing the emission wavelength. The *n* orbital in aggregated **PUH** is much higher than that in the gas phase or aqueous solution and gives the smallest *n*–π* gap among the three systems, which corresponds well to the statistical number of hydrogen bonds (Table S4 and Figure 3b).

As presented by natural transition orbitals (NTOs) with eigenvalue >10^–6^ in Figures S18–S20, simply replacing the boron atom by a carbon–hydrogen or a nitrogen atom does not change the excitation feature of the **PUH**’s photofunctional center, i.e., the S_1_ state with the *n* → π* property is retained. Although the NTOs show that luminescence of **PUH** originates from the *n* → π* transition on C=O, where the boron atom is not directly involved, by replacing boron with carbon (**PUH-C**) and nitrogen (**PUH-N**), the excitation energy of the S_1_ state still shows a difference and **PUH** has the most stable S_1_ state (Table S3). From **PUH-N**, **PUH-C** to **PUH**, a higher *n* orbital level leads to a smaller *n*–π* energy gap, giving a more stable S_1_ excited state, observed as a red-shift in the emission wavelength in the aggregated state (Table S4). By comparison, with B atoms as the electron-deficient center, reducing the electron density of the *n* orbital nearby C=O through a field effect, increases the energy of the *n* orbital, reducing the energy level difference of *n*–π*, finally reducing the S_1_ energy level of **PUH**, especially in the aggregation state (Figure 4).

**Figure 4:**
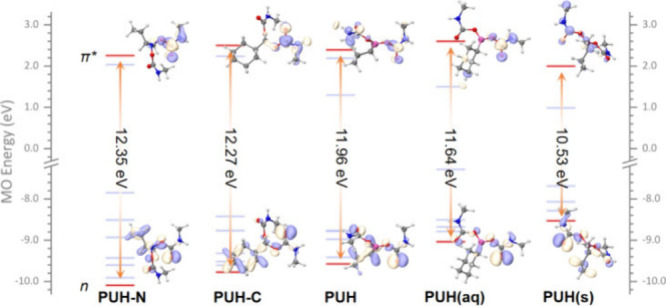
Energy level diagram of **PUH-N**, **PUH-C**, **PUH**, **PUH**(aq), and **PUH**(s).

To explore a practical application of the aggregation-induced luminochromism of **PUH**, we doped solid **PUH** into PMMA in DMF solutions and then prepared **PUH**-PMMA films by evaporation. The emission spectrum and photographs are shown in Figures 5a,c. The amount of PMMA was fixed at 500 mg, while the amount of **PUH** was varied: plastic films with progressive blue to green to orange fluorescence were successfully obtained (Figure 5b). Concentration-dependent color tunability was attributed to the presence of different forms of the **PUH** molecules in the PMMA matrix. That is, isolated **PUH** molecules exist in the low-concentration doped film, and then with increased **PUH** doping, **PUH** gradually aggregates, thus showing an emission color (Figure 5c) similar to **PUH** powder (Figure 1a). In addition, based on the complementary color principle, a film should be obtained with white-light emission by regulating the doping ratio. However, when the amount of **PUH** exceeded 5 mg, the emission of the film was always blue–green–orange, and white light was not observed. The color varied between green and yellow (Figure 5c), and eventually we fixed the amount of **PUH** at 9.4 mg, which seemed most likely to give white light, and in turn regulated the amount of PMMA. After a series of attempts (Figure S21), a white-light-emitting **PUH**-PMMA film was obtained at the ratio of 9.4:440 with Commission Internationale de l’éclaraige (CIE) coordinates (0.29, 0.34) (Figure S22), which are very close to ideal white light (0.33, 0.33).

**Figure 5:**
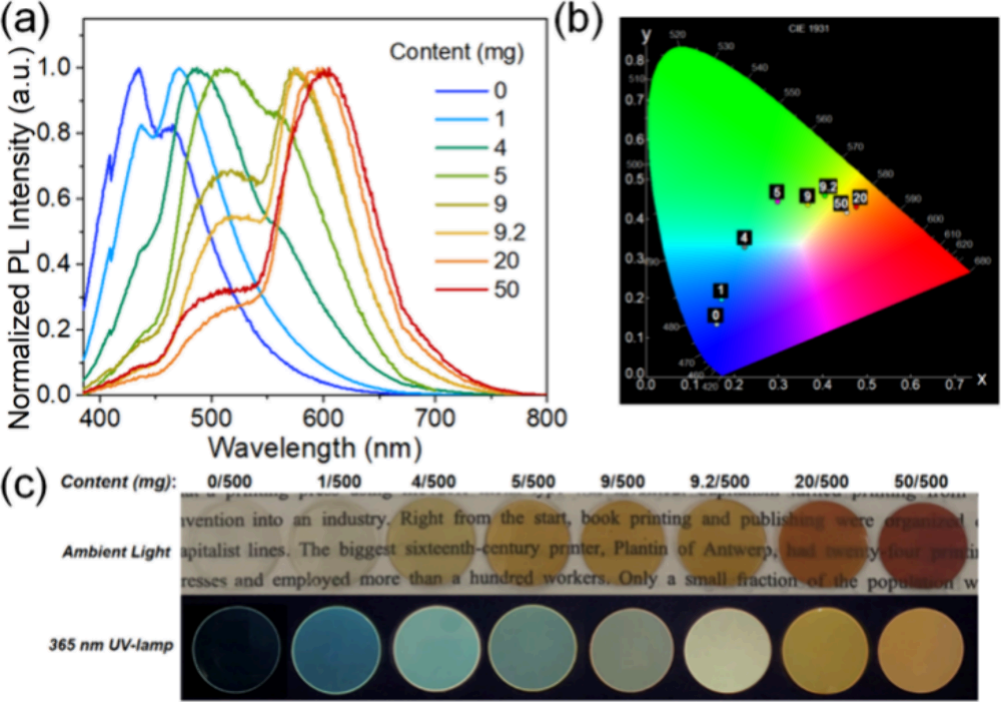
(a) Normalized emission spectra of **PUH**-PMMA films with different doping ratios. (b) CIE diagram of the multicolor emitting films. (c) Corresponding photographs of the films with different doping ratios.

In conclusion, the diversity of emission from a single material is important for multifunctional applications. However, multicolor emission depends strongly on the specific molecular conformation and the precise mode of crystal packing. These properties often cannot be controlled or predicted, and the poor processing properties (high brittleness and low flexibility) of crystalline materials greatly limit their practical applications. In this study, we overcame these problems by exploiting aggregation-induced luminochromism, and by changing the aggregation degree of **PUH** in a PMMA film to obtain multicolor (blue to orange) and white-light-emitting films. Detailed experimental and theoretical calculations show that the final photoluminescent color of the material is closely related to the degrees of polymerization, the strength of the inter/intramolecular interactions, and the final microaggregation morphology of the material. This work advances the exploration of nonconventional polyurethane chromophores with molecular aggregation and provides new insights for the acquisition of solid state and thin film multicolor luminescent materials for practical applications.

## Data Availability

The data associated with this Article is available in the manuscript and Supporting Information files.

## Supporting Information Available

Experimental details; Scheme S1; Figures S1–S22; Tables S1–S4; references (PDF)
