# Supplementary material for: Nonconjugated Polyurethane Derivatives with Aggregation-Induced Luminochromism for Multicolor and White Photoluminescent Films
Source: ACS Macro Lett. 2024 Sep 9;13(10):1226–32. doi: 10.1021/acsmacrolett.4c00534 (PMC11483944; doi:10.1021/acsmacrolett.4c00534)
Supplement: Supplementary file 1 — mz4c00534_si_001.pdf [file mz4c00534_si_001.pdf]

Supporting Information

Non-Conjugated Polyurethane Derivatives with Aggregation-Induced Luminochromism for Multicolor and White Photoluminescent Films

Nan Jiang,[a] Ya-Jie Meng,[b] Chang-Yi Zhu,[a] Ke-Xin Li,[a] Xin Li,[a] Yan-Hong Xu,[*a] Jia-Wei Xu,[*b] and Martin R. Bryce[*c]

[a] Key Laboratory of Preparation and Applications of Environmental Friendly Materials, Key Laboratory of Functional Materials Physics and Chemistry of the Ministry of Education (Jilin Normal University), Changchun, 130103, China.
[b] Ministry-of-Education Key Laboratory of Numerical Simulation of Large-Scale Complex System (NSLSCS) and School of Chemistry and Materials Science, Nanjing Normal University, Nanjing 210023, China.
[c] Department of Chemistry, Durham University, Durham, DH1 3LE, UK
E-mail: xuyh198@163.com; jwxu_njnu@sina.com; m.r.bryce@durham.ac.uk

Contents:

1. Experimental details

2. Methodology, synthesis and structural characterization

3. Photophysical properties and theoretical calculations

4. References

1. Experimental details

General

The UV-vis absorption spectra were recorded on a Shimadzu UV-3100 spectrophotometer. The fluorescence spectra and lifetimes (τ) were recorded on an Edinburgh Instruments FLS-1000 spectrometer. The fluorescence and fluorescence quantum yields were recorded using an Edinburgh Instruments FLS-1000 spectrometer. ^{1}H NMR spectra were recorded on a Varian 500 MHz spectrometer. The ^{1}H NMR spectra were referenced internally to the residual proton resonance in DMSO-d_6 (δ 2.5 ppm). The molecular weights of the polyurethane samples were determined by gel permeation chromatography (GPC) on a Waters 410 instrument with monodispersed polystyrene as the reference and THF as the eluent at 35 °C. Scanning electron microscope (SEM) images were obtained using a JEOL model JSM-6700 instrument operating at an accelerating voltage of 5.0 kV.

2. Methodology

2.1 Molecular Dynamics

Two molecular dynamics systems, including (1) 50 model molecules of **PUH** (m = 2 and n = 2) to describe polymer cluster environment, and (2) 1 model molecule of **PUH** surrounded by 1960 water molecules to describe aqueous environment, were built by Packmol program. The model molecular **PUH** was optimized at the PBE0-D3(BJ)/def2-SVP level of theory[1-4] using Gaussian 16 (Revision C.01)[5] and no imaginary frequency was checked by frequency calculation. The restrained electrostatic potential (RESP) atomic charges were generated by Multiwfn.[6] Force field parameters were adopted from generalized Amber force field (GAFF).[7]

Molecular dynamics (MD) simulations were performed using the GROMACS (version 2022.5) package[9] and topology file and forcefield parameters were created by Sobtop.[8] The long-range electrostatic interactions were handled by the particle-mesh Ewald (PME) method and the cutoff value of van der Waals interactions was set to 10.0 Å.[10] After energy minimization, the three systems were heated up from 0 K to 300 K in the 1.0 ns simulations. Subsequently, the 50.0 ns MD simulations (MD1) were conducted in the NPT ensemble at 300 K using the v-rescale thermostat method[11] and the Berendsen.[12] Next, both systems were heated up to synthetic temperature 403 K, with the 50.0 ns of MD simulations (MD2). Finally, the temperature of the three systems dropped to 300 K and unrestrained MD simulations for 50.0 ns (MD3) were performed.

2.2 QM/MM Calculations

Natural transition orbitals (NTO) were calculated under NEVPT2(6,6)/def2-TZVP/MM level of theory. Electron-hole distribution was obtained based on NTO results. The snapshots were taken from MD3 and optimized under CAM-B3LYP-D3/def2-SVP level of theory by Tcl-Chemshell program (version 3.7.1),[13] where QM region was treated by ORCA program (version 5.0.4)[14] and MM region was treated by DL_poly program[15] with electrostatic embedding scheme. Active spaces used for multi-reference calculation was selected by standard workflow of MOKIT[16] based on the optimized structure, including: 1). RHF/def2-TZVP single point calculation was performed with background charges and wavefunction was checked to be stable by Gaussian16; 2). CIS/def2-TZVP calculation with background charges was performed for lowest 7 states based on previous RHF wavefunction and all excitation components with contribution larger than 10^{-5} were considered and 3). Active spaces were determined based on NTO obtained in CIS calculation.[17, 18] NEVPT2/MM calculation was performed by PySCF package (version 2.5.0).[19]

2.3 QM Calculation

To investigate to function of boron atom, three model system were designed based on QM/MM results, as displayed in Scheme S1. Boron atom in the photofunctional center suggested by QM/MM results was replaced by carbon and nitrogen atom, respectively, to obtain PUH-C and PUH-N analogues for investigation. The geometry optimization and frequency calculation of these QM-only systems were performed by Gaussian16 program under M06-2X-D3/def-SVP level of theory. Multireference calculations were performed using similar approaches described in previous section.

Synthesis

Reagents for synthesis were purchased from commercial suppliers. Cyclohexylboronic acid and (from Adamas and Energy Chemical) was recrystallized from the ethyl alcohol before use.

Synthesis of PUH

A mixture of cyclohexylboronic acid (1.676 g, 13.1 mmol), anhydrous DMSO (8 mL), isophorone diisocyanate (3.034 g, 13.65 mmol) and 1,4-diazabicyclooctane triethylenediamine (DABCO) (0.059 g, 0.525 mmol) was added to a dried two-neck round-bottom flask. The

solution was heated at 130 °C for 8 h under nitrogen atmosphere. After that time the clear solution had become significantly viscous, indicating the occurrence of polymerization. The crude product was dissolved in chloroform and then reverse precipitated from excess diethyl ether. The product was dried under vacuum for 24 h to obtain **PUH** (2.544 g). Yield: 54%.

Synthesis of P1

A mixture of cyclohexylboronic acid (1.006 g, 7.86 mmol), anhydrous THF (3 mL), anhydrous DMSO (3 mL), isophorone diisocyanate (1.821 g, 8.19 mmol) and 1,4-diazabicyclooctane triethylenediamine (DABCO) (0.035 g, 0.315 mmol) was added to a dried two-neck round-bottom flask. The solution was heated at 100 °C for 7 h under nitrogen atmosphere. After that time the clear solution had become significantly viscous, indicating the occurrence of polymerization. The crude product was dissolved in chloroform and then reverse precipitated from excess diethyl ether. The product was dried under vacuum for 24 h to obtain **P1** (1.75 g). Yield: 62%.

Synthesis of P2

A mixture of cyclohexylboronic acid (1.006 g, 7.86 mmol), anhydrous THF (3 mL), anhydrous DMSO (3 mL), isophorone diisocyanate (1.821 g, 8.19 mmol) and 1,4-diazabicyclooctane triethylenediamine (DABCO) (0.035 g, 0.315 mmol) was added to a dried two-neck round-bottom flask. The solution was heated at 80 °C for 7 h under nitrogen atmosphere. After that time the clear solution had become significantly viscous, indicating the occurrence of polymerization. The crude product was dissolved in chloroform and then reverse precipitated from excess diethyl ether. The product was dried under vacuum for 24 h to obtain **P2** (1.64 g). Yield: 58%.

Synthesis of P3

A mixture of cyclohexylboronic acid (1.676 g, 13.1 mmol), anhydrous DMSO (8 mL), isophorone diisocyanate (3.034 g, 13.65 mmol) and 1,4-diazabicyclooctane triethylenediamine (DABCO) (0.059 g, 0.525 mmol) was added to a dried two-neck round-bottom flask. The solution was heated at 125 °C for 23 h under nitrogen atmosphere. After that time the clear solution had become significantly viscous, indicating the occurrence of polymerization. The crude product was dissolved in chloroform and then reverse precipitated from excess diethyl ether. The product was dried under vacuum for 24 h to obtain **P3** (2.69 g). Yield: 57%.

Synthesis of PUC

A mixture of 1,4-cyclohexanediol (0.609 g, 5.24 mmol), anhydrous DMSO (6 mL), isophorone diisocyanate (1.213 g, 5.46 mmol) and 1,4-diazabicyclooctane triethylenediamine (DABCO) (0.024 g, 0.21 mmol) was added to a dried two-neck round-bottom flask. The solution was heated at 140°C for 21 h under nitrogen atmosphere. After that time the clear solution had become significantly viscous, indicating the occurrence of polymerization. The crude product was dissolved in chloroform and then reverse precipitated from excess diethyl ether. The product was dried under vacuum for 24 h to obtain **PUC** (1.27 g). Yield: 70%.

Preparation method of photoluminescent PUH/PMMA films

PMMA powder (0.5 g) was stirred into DMF (10 mL) until dissolved. Different weights of **PUH** powder were dissolved in DMF (2 mL). The two solutions were mixed in a beaker which was placed on a heating table (140 °C) to slowly evaporate the solvent. The resulting film was removed from the beaker and a tablet press was used to cut a circle of the film.

2. Structural characterization

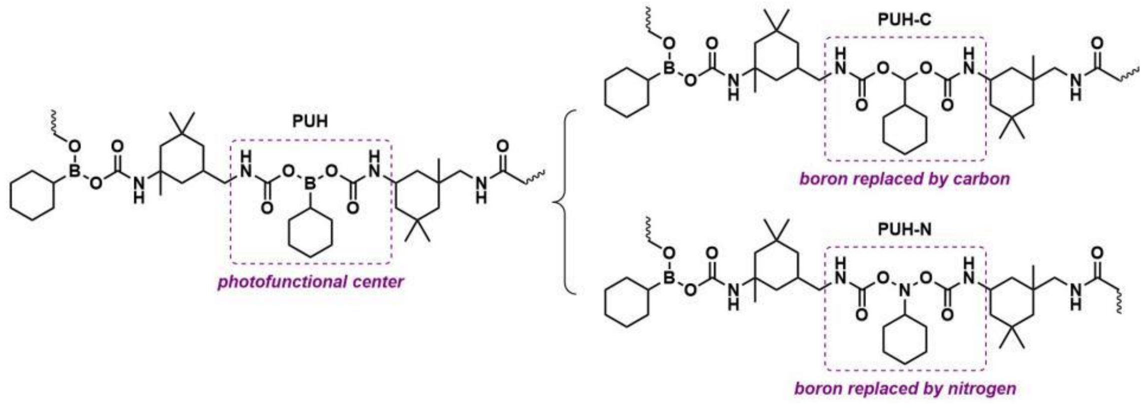

Scheme S1. Photofunctional center of **PUH** suggested by QM/MM calculations and model systems **PUH-C** and **PUH-N** designed by replacing boron atom in **PUH** by carbon-hydrogen and nitrogen atoms.

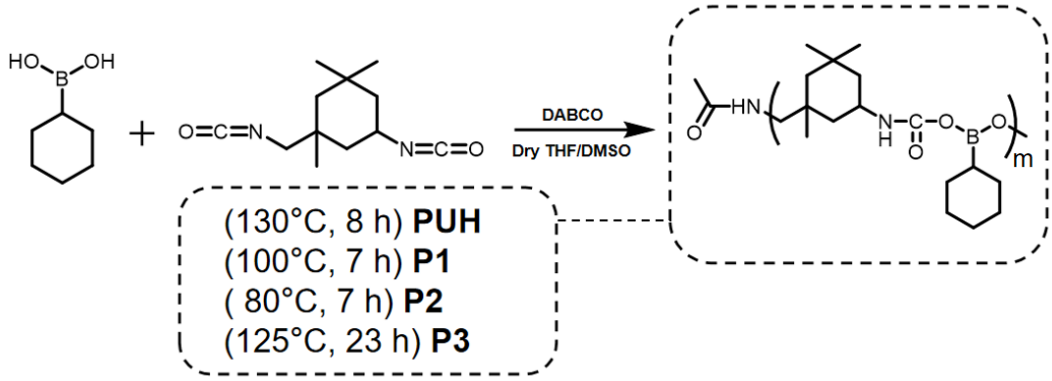

Figure S1. Synthetic route to the polyurethane derivative **PUH**, **P1**, **P2** and **P3**.

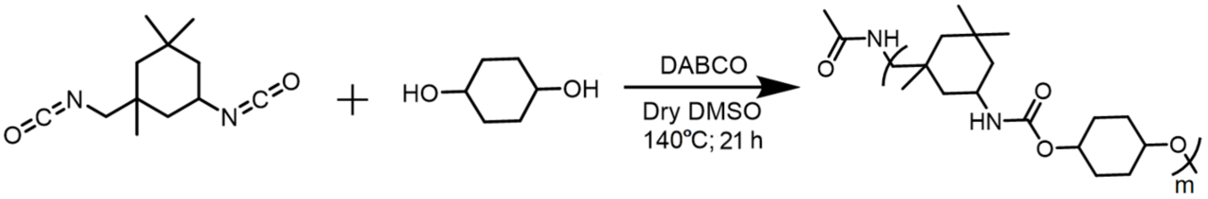

Figure S2. Synthetic route to the polyurethane derivative **PUC**.

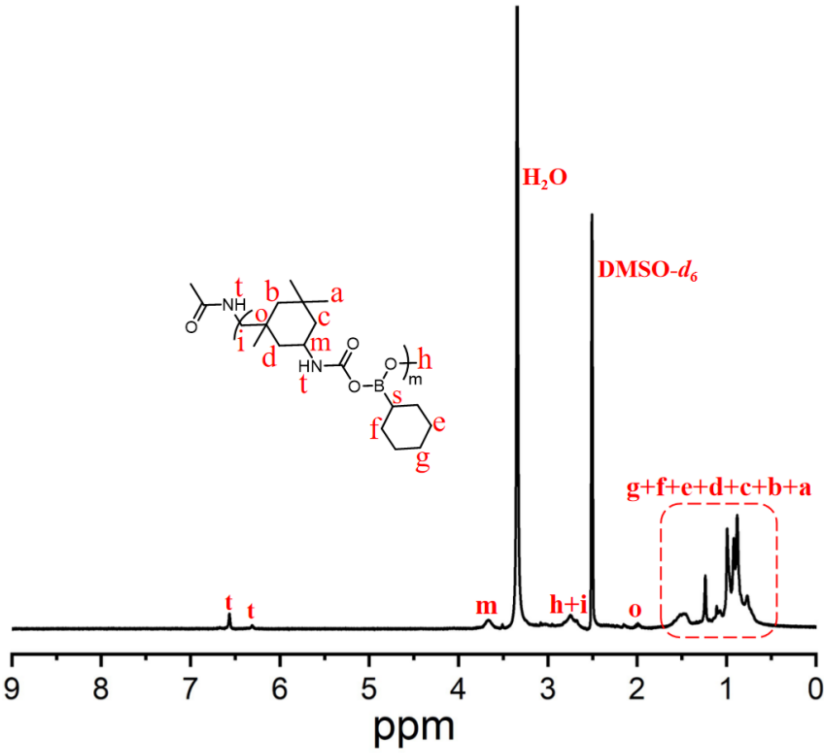

Figure S3. ^{1}H NMR spectrum of **PUH** in DMSO-d_6.

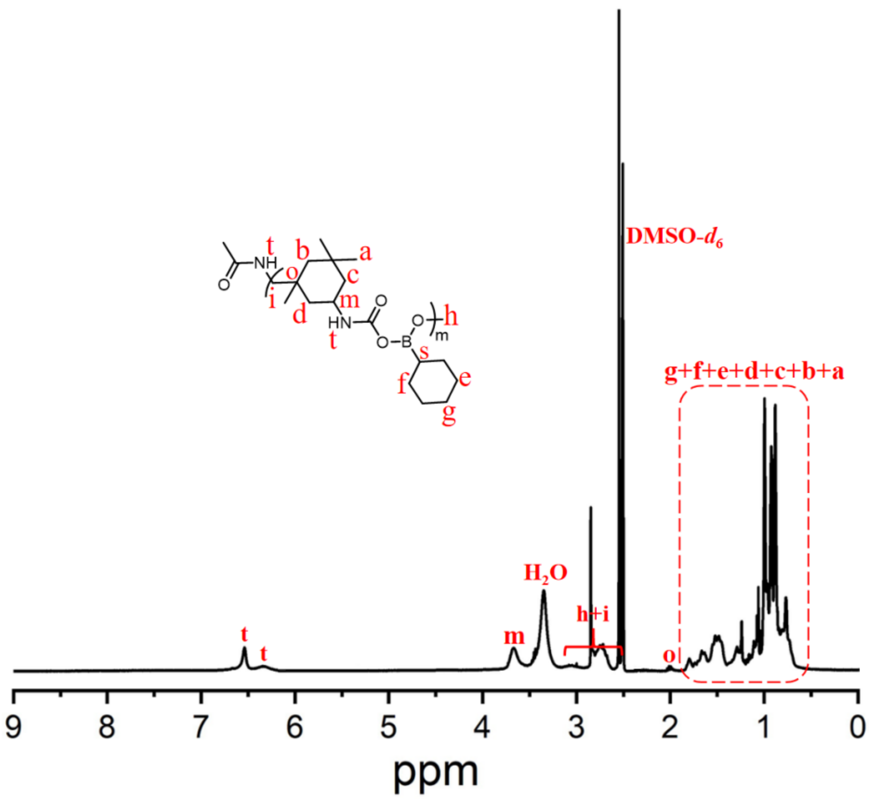

Figure S4. ^{1}H NMR spectrum of **P1** in DMSO-d_6.

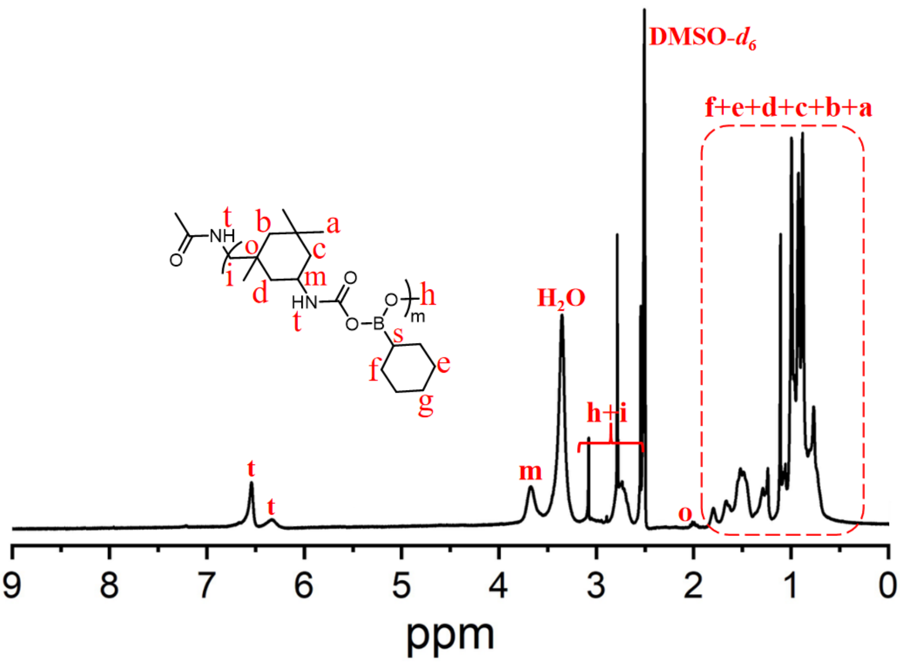

Figure S5. ^{1}H NMR spectrum of **P2** in DMSO-d_6.

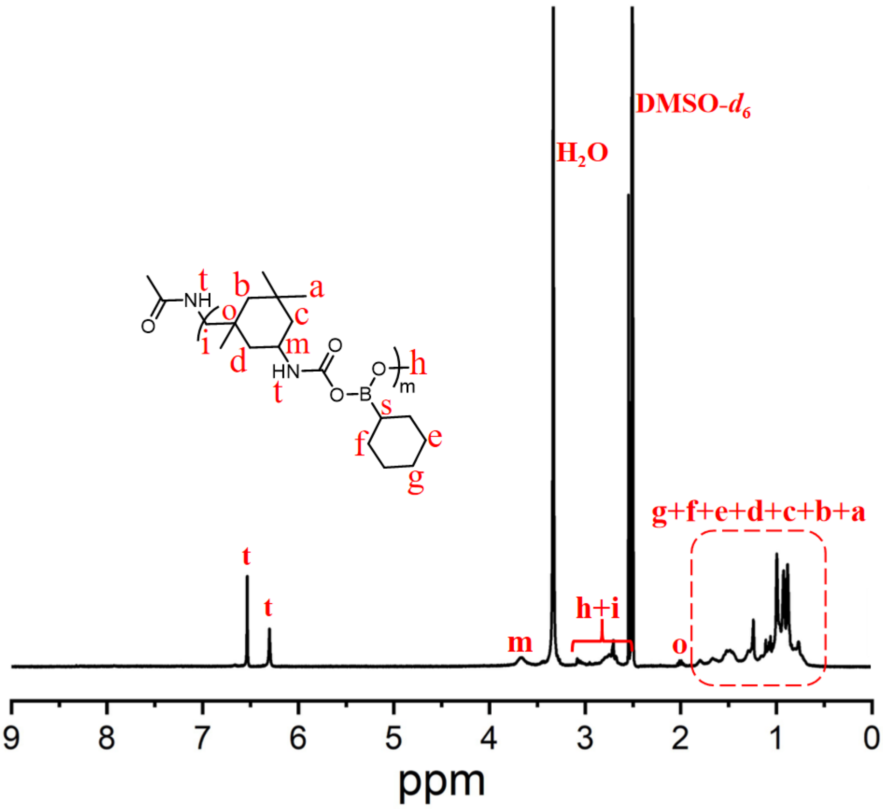

Figure S6. ^{1}H NMR spectrum of **P3** in DMSO-d_6.

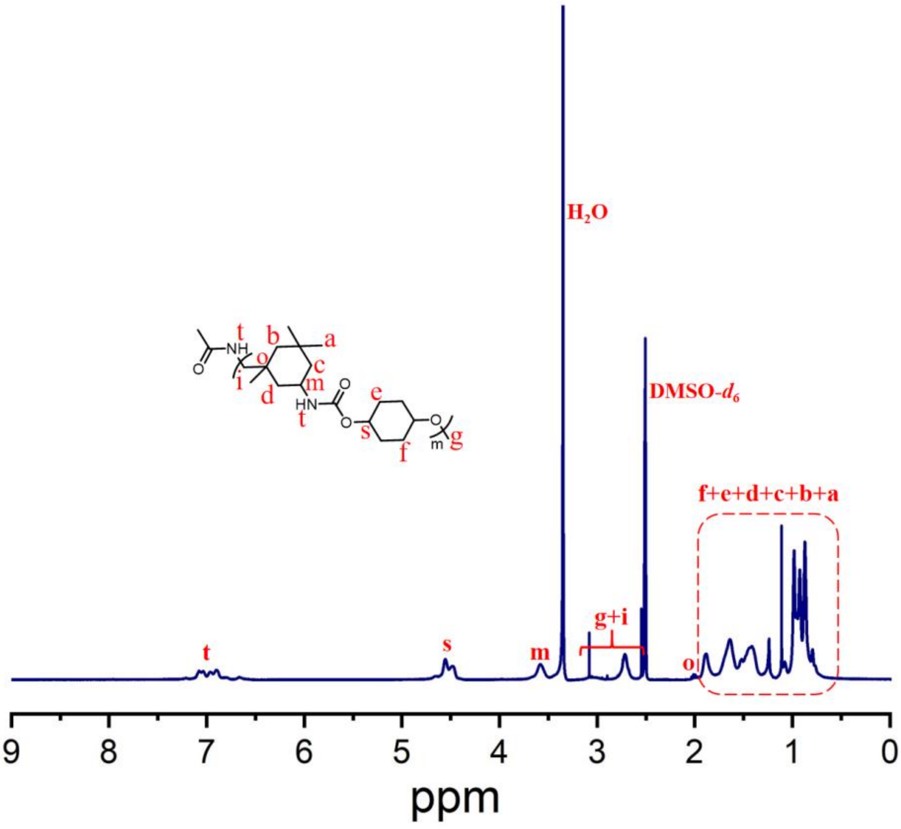

Figure S7. ^{1}H NMR spectrum of **PUC** in DMSO-d_6.

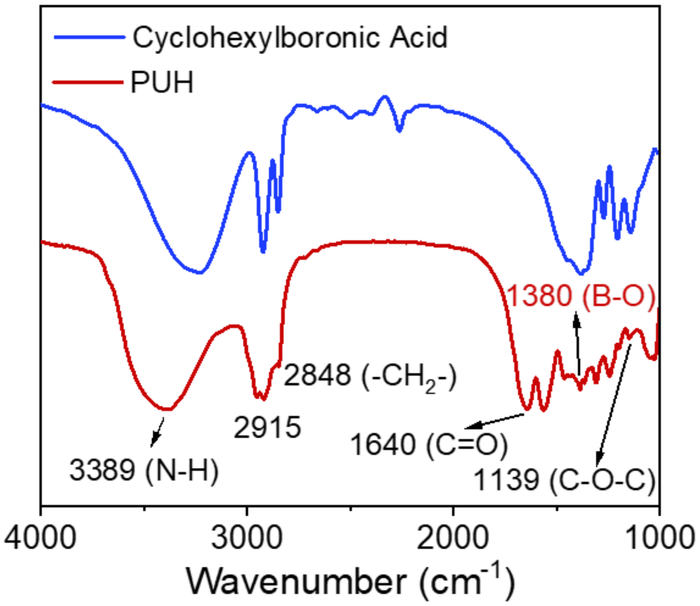

Figure S8. FT-IR spectra of cyclohexylboronic acid monomer and **PUH** powder.

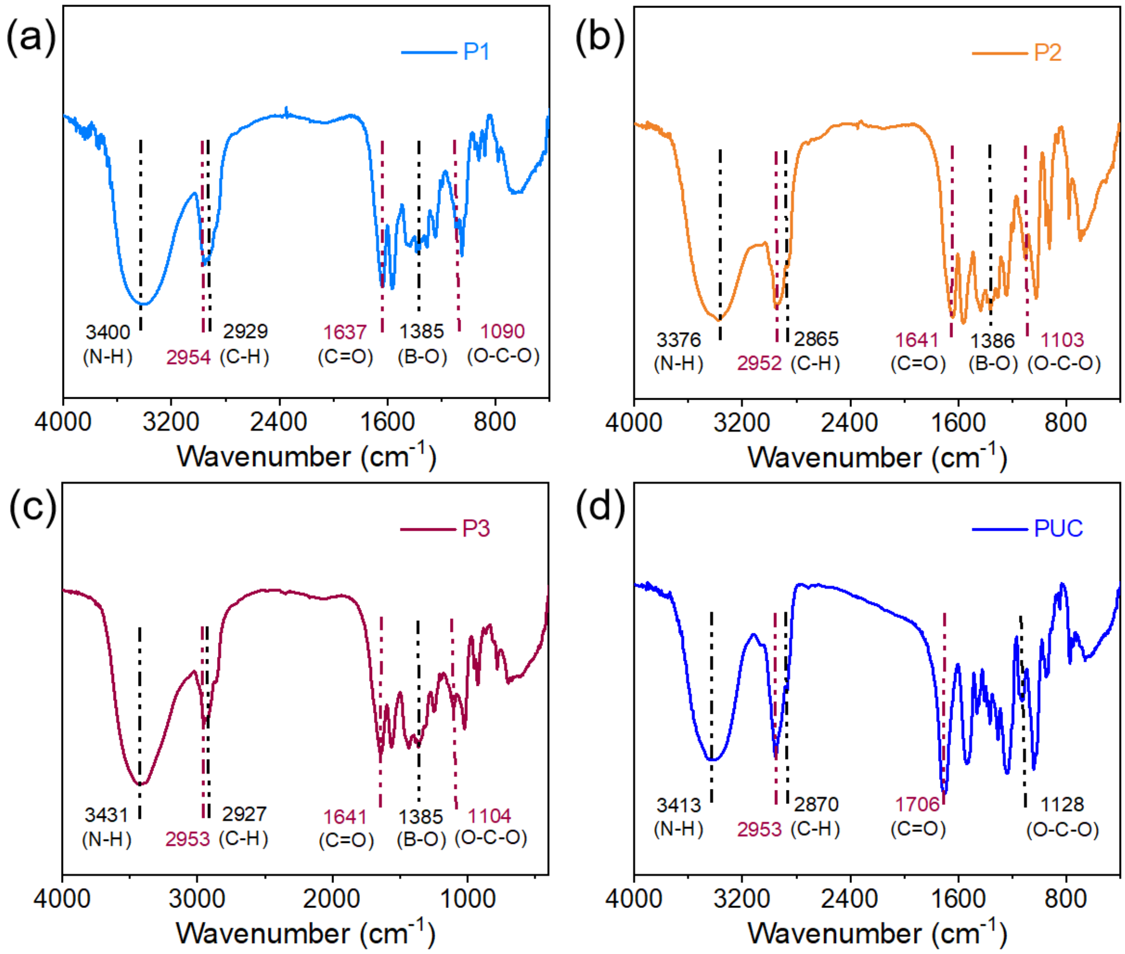

Figure S9. FT-IR spectrum of (a) **P1**, (b) **P2**, (c) **P3** and (d) **PUC** in solid state.

3. Photophysical properties and theoretical calculations

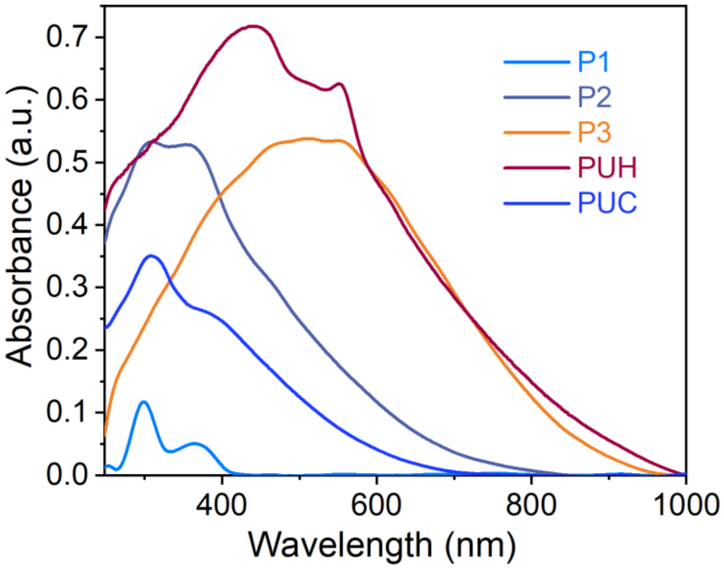

Figure S10. Absorption spectrum of **P1**, **P2**, **P3**, **PUH** and **PUC** powders.

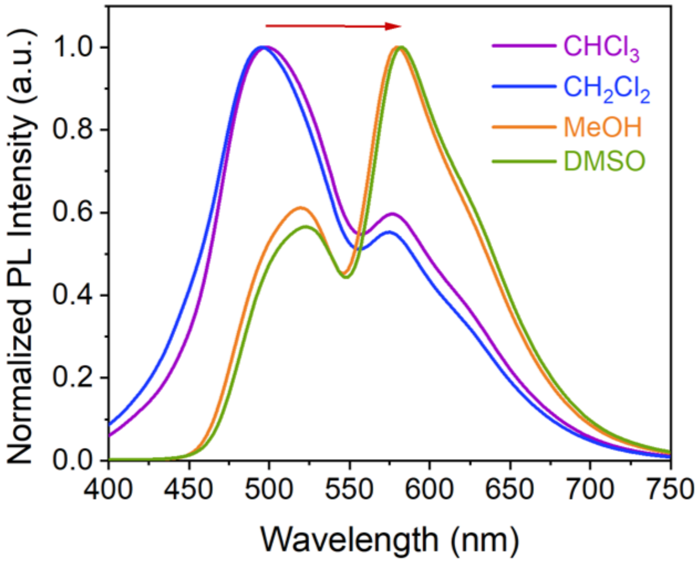

Figure S11. Normalized emission spectra of **PUH** in trichloromethane ($CHCl_3$), dichloromethane (CH_2Cl_2), methanol (MeOH) and dimethyl sulfoxide (DMSO) solvent (3 mg mL^{-1}).

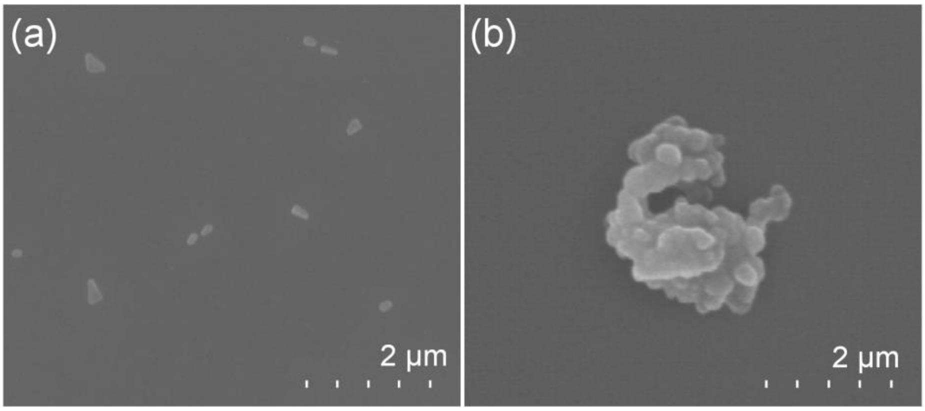

Figure S12. SEM images of 20 mg mL^{-1} (a) cyclohexylboronic acid, (b) **PUH** dispersed in DMSO solution.

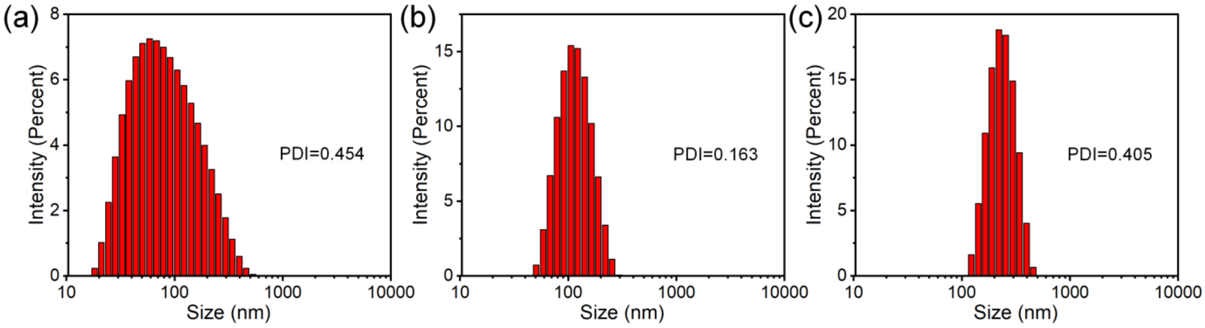

Figure S13. DLS particle size map of **PUH** (a) 0.1 mg mL^{-1}, (b) 1 mg mL^{-1} and (c) 5 mg mL^{-1} in pure DMSO solution.

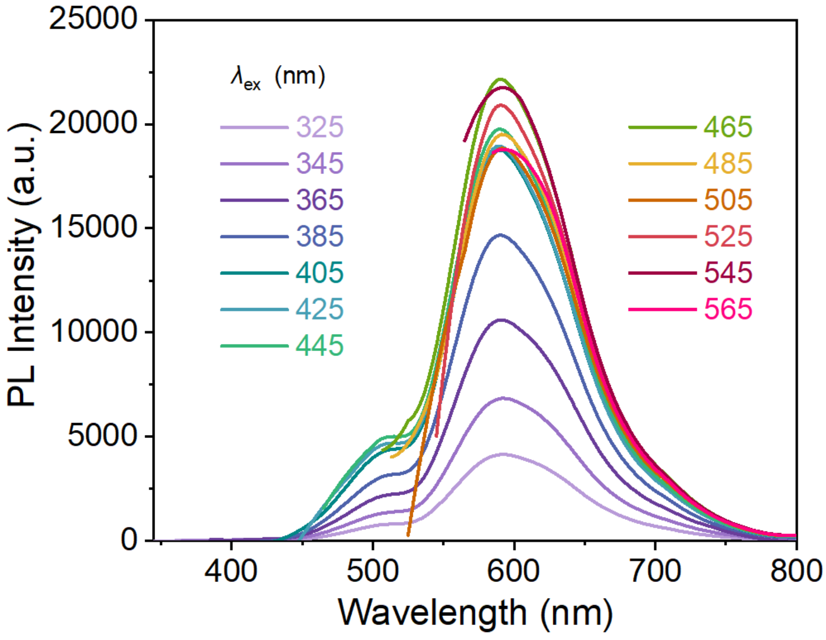

Figure S14. PL spectra of **PUH** powder at varying λ_{ex}.

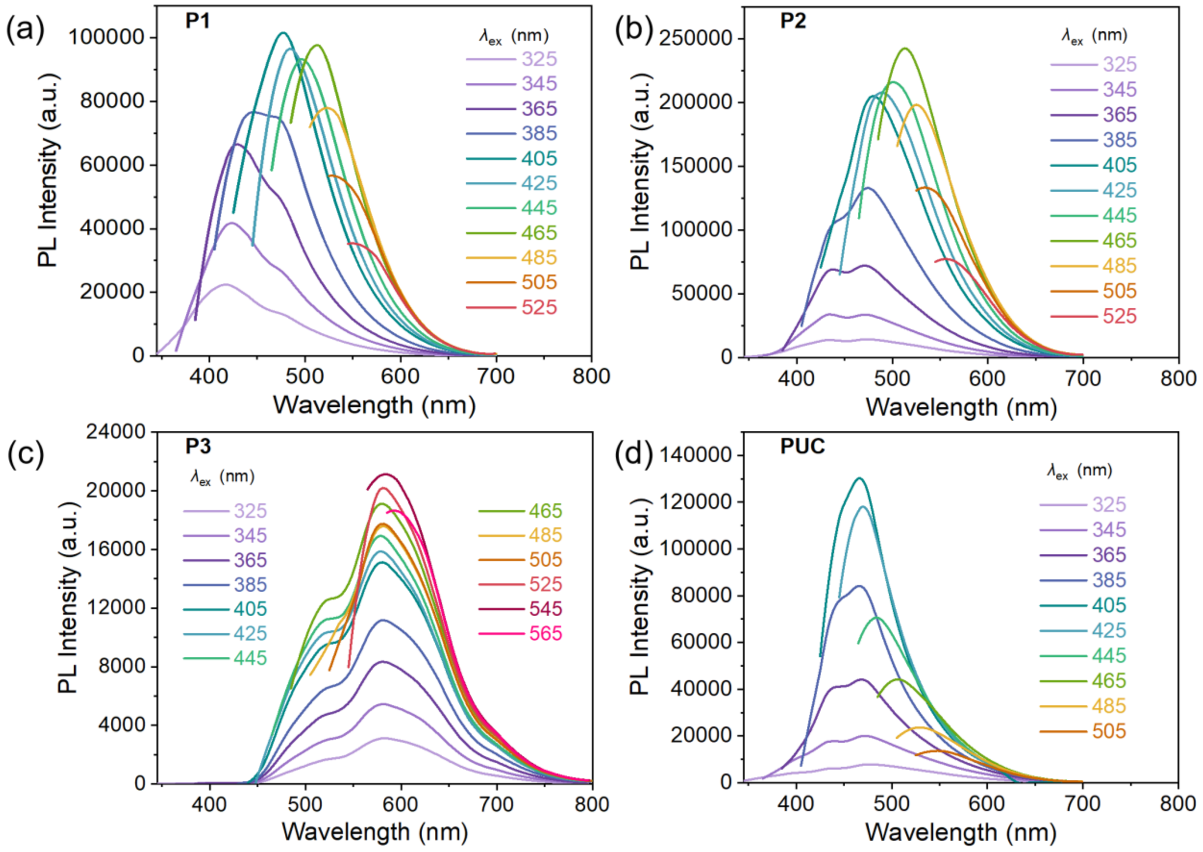

Figure S15. Emission spectra of (a) **P1**, (b) **P2**, (c) **P3** and (d) **PUC** powders with different excitation wavelengths at room temperature.

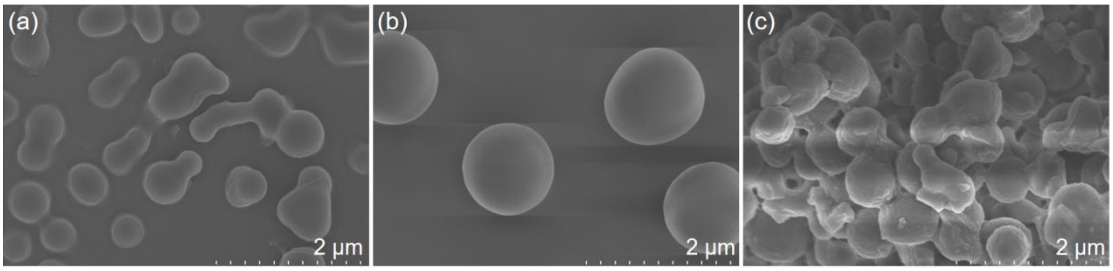

Figure S16. SEM images of contrast (a) **P1**, (b) **P2** and (c) **P3**/DMSO solution (20 mg mL^{-1}).

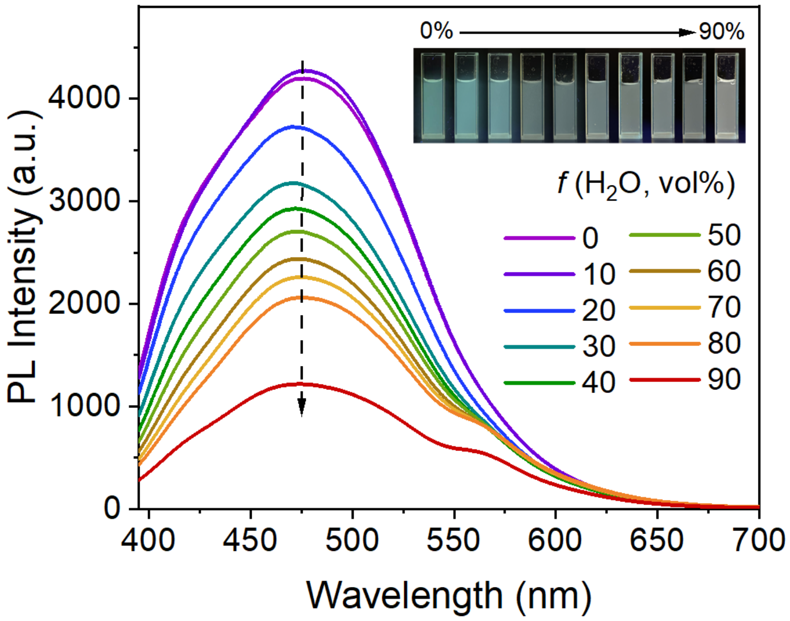

Figure S17. Emission spectra of 1 mg mL^{-1} **PUH**/cyclohexylboronic acid monomer (1:1 w/w) in DMSO–water mixtures with different water fractions (0–90%) at room temperature. Insets: photographs of these mixture under 365 nm UV illumination.

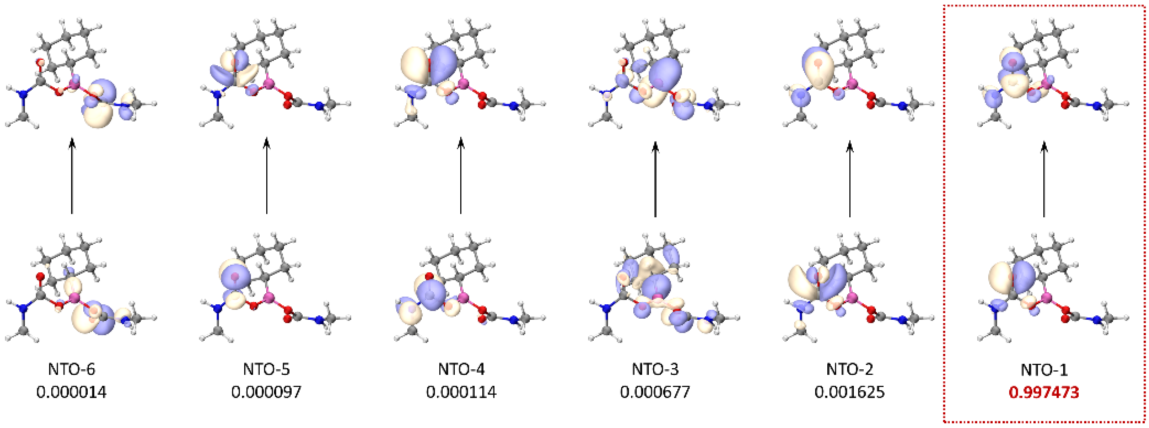

Figure S18. S_1 state excitation feature of **PUH** photofunctional center revealed by natural transition orbitals (isovalue: 0.050 a.u.).

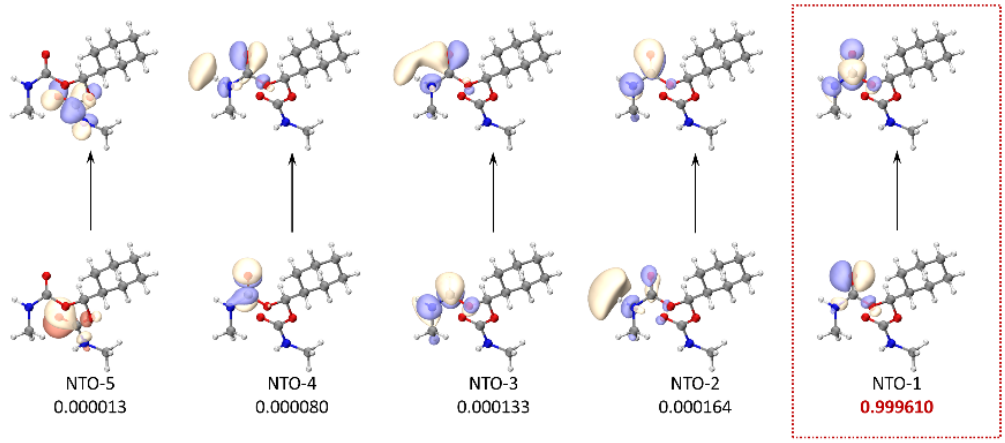

Figure S19. S_1 state excitation feature of **PUH-C** photofunctional center revealed by natural transition orbitals (isovalue: 0.050 a.u.).

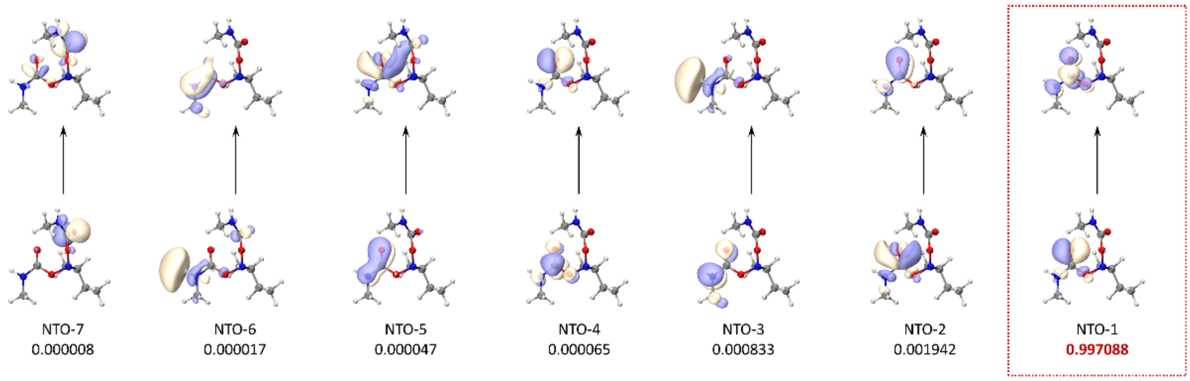

Figure S20. S_1 state excitation feature of **PUH-N** photofunctional center revealed by natural transition orbitals (isovalue: 0.050 a.u.).

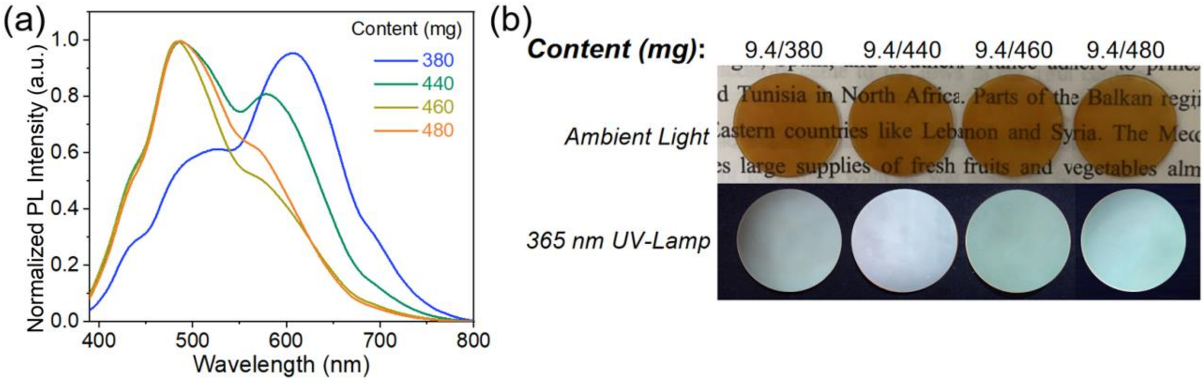

Figure S21. (a) Normalized emission spectra of **PUH**/PMMA film with different mixing ratios. (b) The corresponding photographs of the films.

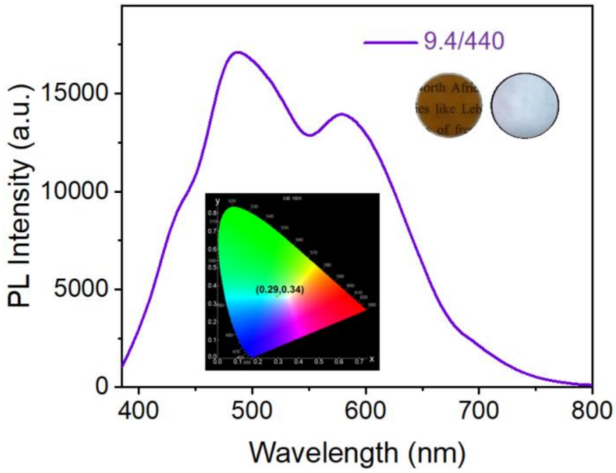

Figure S22. PL spectra of **PUH**/PMMA film (9.4/440 mg). Insert: Photographs of the film under daylight (left) and 365 nm UV light (right); CIE diagram of the white-light-emitting film.

Table S1. Optical properties of **PUH**, **P1**, **P2**, **P3** and **PUC**.

	λ_{abs}	$\lambda_{em}{}^{a}$	LT^{a}	QY
**PUH**	451 nm	591 nm	1.27 ns (591 nm)	5.8%
**P1**	299 nm	428 nm	5.18 ns (428 nm)	8.5%
**P2**	307 nm	470 nm	3.79 ns (470 nm)	3.5%
**P3**	518 nm	580 nm	2.03 ns (580 nm)	1.6%
**PUC**	307 nm	469 nm	2.25 ns (469 nm)	3.1%

^{a} $\lambda_{ex=}$365 nm

Table S2. Molecular weight data of the PUs from GPC.

	**PUH**	**P1**	**P2**	**P3**
M_n	2352	5338	5175	4079
M_w	3570	6005	5762	4236
M_P	1146	5102	4846	3628
**PD**	1.52	1.12	1.11	1.04

Table S3. Excitation energy (eV) and oscillator strength (a.u.) for the lowest five excited states obtained by NEVPT2(/MM) calculations.

State No.	**PUH-N**	**PUH-C**	**PUH** ^{a}	**PUH**(aq) ^{b}	**PUH**(s) ^{c}
S_1	**7.335 (0.0044)**	**7.385 (0.0003)**	**6.996 (0.0385)**	**6.978 (0.0563)**	**4.609 (0.0238)**
S_2	7.655 (0.0002)	7.477 (0.0001)	7.053 (0.0003)	6.989 (0.0276)	6.183 (0.2142)
S_3	8.271 (0.0004)	8.770 (0.0336)	7.278 (0.0018)	7.036 (0.0873)	6.625 (0.0064)
S_4	8.730 (0.1045)	8.907 (0.0197)	8.518 (0.1262)	7.278 (0.0056)	6.693 (0.0378)
S_5	9.586 (0.4498)	9.397 (0.3913)	8.707 (0.2554)	7.357 (0.0006)	7.964 (0.0908)

^{a} Denotes QM-only model system of **PUH** including photofunctional center; ^{b} **PUH** ($m = 2$ and $n = 2$) in water box; ^{c} Cluster system including 50 **PUH** ($m = 2$ and $n = 2$) molecules.

Table S4. Orbital energies (eV).

	**PUH-N**	**PUH-C**	**PUH**	**PUH**(aq)	**PUH**(s)
HOMO	-7.849352	-8.422172	-8.766414	-7.283976	-7.686486
LUMO	2.034657	2.238958	1.299174	1.499582	0.987717
HOMO-LUMO	9.884009	10.661130	10.065588	8.783558	8.674203
**C=O *n***	**-10.093008**	**-9.773519**	**-9.573651**	**-9.037613**	**-8.528325**
C=O π*	2.254002	2.500592	2.390875	2.600866	1.999956
n*-π	**12.347010**	**12.274111**	**11.964526**	**11.638479**	**10.525281**

4. References

(1) Grimme, S. Semiempirical GGA-type Density Functional Constructed with a Long-Range Dispersion Correction. *J. Comput. Chem.* **2006**, *27*, 1787-1799.

(2) Perdew, J. P.; Burke, K.; Ernzerhof, M. Generalized Gradient Approximation Made Simple. *Phys. Rev. Lett.* **1996**, *77*, 3865-3868.

(3) Papajak, E.; Leverentz, H. R.; Zheng, J.; Truhlar, D. G. Efficient Diffuse Basis Sets: cc-pVxZ+ and maug-cc-pVxZ. *J. Chem. Theory Comput.* **2009**, *5*, 3330-3330.

(4) Weigend, F. Accurate Coulomb-fitting basis sets for H to Rn. *Phys. Chem. Chem. Phys.* **2006**, *8*, 1057-1065.

(5) Frisch, M. J.; Trucks, G. W.; Schlegel, H. B.; Scuseria, G. E.; Robb, M. A.; Cheeseman, J. R.; Scalmani, G.; Barone, V.; Petersson, G. A.; Nakatsuji, H. et al. Gaussian 16 Rev. C. 01. *Wallingford*, *CT*, **2016**.

(6) Lu, T.; Chen, F. Multiwfn: A Multifunctional Wavefunction Analyzer. *J. Comput. Chem.* **2012**, *33*, 580-592.

(7) Sprenger, K. G.; Jaeger, V. W.; Pfaendtner, J. The General AMBER Force Field (GAFF) Can Accurately Predict Thermodynamic and Transport Properties of Many Ionic Liquids. *J. Phys. Chem. B* **2015**, *119*, 5882-5895.

(8) Hess, B.; Kutzner, C.; Van Der Spoel, D.; Lindahl, E. GROMACS 4: Algorithms for Highly Efficient, Load-Balanced, and Scalable Molecular Simulation. *J. Chem. Theory Comput.* **2008**, *4*, 435-447.

(9) Tian, L. *Sobtop*, 1.0(dev3.1).

(10) Darden, T.; York, D.; Pedersen, L. Particle Mesh Ewald: An *N*·Log(*N*) Method for Ewald Sums in Large Systems. *J. Chem. Theory Comput.* **1993**, *98*, 10089-10092.

(11) Bussi, G.; Donadio, D.; Parrinello, M. Canonical Sampling Through Velocity Rescaling. *J. Chem. Phys.* **2007**, *126*, 014101.

(12) Parrinello, M.; Rahman, A. Polymorphic Transitions in Single Crystals: A New Molecular Dynamics Method. *J. Appl. Phys.* **1981**, *52*, 7182-7190.

(13) Metz, S.; Kästner, J.; Sokol, A. A.; Keal, T. W.; Sherwood, P. ChemShell-a Modular Software Package for QM/MM Simulations. *WIREs Comput. Mol. Sci.* **2014**, *4*, 101-110.

(14) Neese, F. Software Update: The ORCA Program System-Version 5.0. *WIREs Comput. Mol. Sci.* **2022**, *12*, e1606.

(15) Smith, W.; Yong, C. W.; Rodger, P. M. DL_POLY: Application to Molecular Simulation. *Mol. Simulat.* **2002**, *28*, 385-471.

(16) Zou, J. Molecular Orbital Kit (MOKIT). https://gitlab.com/jxzou/mokit (accessed 2023.11.01).

(17) Fales, B. S.; Shu, Y.; Levine, B. G.; Hohenstein, E. G. Complete Active Space Configuration Interaction from State-Averaged Configuration Interaction Singles Natural Orbitals: Analytic First Derivatives and Derivative Coupling Vectors. *J. Chem. Phys.* **2017**, *147*, 094104.

(18) Shu, Y.; Hohenstein, E. G.; Levine, B. G. Configuration Interaction Singles Natural Orbitals: An Orbital Basis for An Efficient and Size Intensive Multireference Description of Electronic Excited States. *J. Chem. Phys.* **2015**, *142*, 024102.

(19) Sun, Q.; Berkelbach, T. C.; Blunt, N. S.; Booth, G. H.; Guo, S.; Li, Z.; Liu, J.; McClain, J. D.; Sayfutyarova, E. R.; Sharma, S. Wouters, S.; Chan, G. K. PySCF: the Python-based Simulations of Chemistry Framework. *WIREs Comput. Mol. Sci.* **2018**, *8*, e1340.
